# Treatment and long-term follow-up of pediatric patients with hypocomplementemic urticarial vasculitis syndrome (HUVS): a case-based review

**DOI:** 10.1007/s10067-025-07509-6

**Published:** 2025-06-02

**Authors:** Özen Taş, Fatma Aydın, Zeynep Birsin Özçakar

**Affiliations:** 1 Division of Pediatric Rheumatology, Department of Pediatrics, Ankara University School of Medicine, Ankara, Turkey; 2 Division of Pediatric Nephrology, Department of Pediatrics, Ankara University School of Medicine, Ankara, Turkey

**Keywords:** DNASE1L3 deficiency, Hypocomplementemic urticarial vasculitis, Renal involvement

## Abstract

Hypocomplementemic urticarial vasculitis syndrome (HUVS) is a rare, severe form of urticarial vasculitis. It is characterized by persistent hypocomplementemia, chronic urticarial vasculitic lesions, and severe multiorgan involvement. Herein, we present long-term follow-up of two siblings diagnosed with HUVS at early ages, who were found to have DNASE1L3 mutations, and their subsequent 20-year follow-up. While one of the siblings developed lupus nephritis, the other exhibited vasculitic renal involvement. The patients received various treatments, with rituximab proving most effective in the long term. The present study contributes to the existing body of literature on pediatric HUVS, which, to the best of our knowledge, has been described in 28 cases. Renal involvement was present in 82% of patients, and lupus nephritis was most common in patients with renal pathology. Patients received many different treatments. Two patients died, and five patients developed end-stage renal failure. However, it should be noted that follow-up was not conducted in 39% of these patients and the follow-up period was very short for the remaining patients.

## Introduction

Hypocomplementemic urticarial vasculitis syndrome (HUVS) is a rare and distinct type of specific autoimmune disease with multiorgan involvement. The condition was first described by McDuffie et al. in 1973 on the case presentation of four patients with recurrent urticarial lesions and reduced serum complement levels [1]. The disease is characterized by urticaria with persistent hypocomplementemia and is associated with several systemic findings, including leukocytoclastic vasculitis, severe angioedema, pulmonary involvement, arthritis, arthralgia, glomerulonephritis (GN), and recurrent abdominal pain [1]. The majority of patients are Caucasian, with a female-to-male ratio of approximately 8:1. The disease typically manifests in the fourth decade of life [2, 3].

The diagnosis of HUVS is based on the Schwartz criteria, which include both clinical and laboratory findings: the presence of chronic recurrent urticaria for more than 6 months, in addition to hypocomplementemia (which are major criteria), and the presence of at least two minor criteria, namely leukocytoclastic vasculitis on skin biopsy, arthralgias and arthritis, glomerulonephritis, uveitis or episcleritis, abdominal pain, and a positive C1q antibody test [4]. Despite extensive research, the exact pathophysiology of HUVS remains unknown [5]. Therefore, there is no clear consensus on long-term treatment, especially for patients with resistant and severe disease.

Although HUVS is often idiopathic, there have been reports of a potential association with specific infections, neoplastic processes, and autoimmune disorders such as Sjögren’s syndrome and systemic lupus erythematosus (SLE), as well as a limited number of familial cases [6]. Approximately 50% of HUVS patients are diagnosed with SLE [7].

Besides environmental factors, genetic factors are thought to play a role in the etiology of HUVS. The genetic cause of HUVS was reported for the first time in 2013 [8]. Two of these children are still alive and are being followed up in our clinic. Here, we present the long-term follow-up, treatments, and treatment responses of the two siblings whom we have followed for about 20 years. Moreover, all pediatric patients in the literature were reviewed in this respect.

## Case presentations

### Case 1

**Fig. 1:**
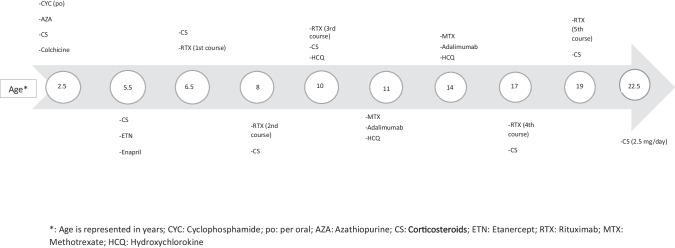
Treatment history of Case 1

A 2.5-year-old girl was accepted to our hospital in 2005 with a history of weakness, recurrent urticaria-like lesions, intermittent fever, and joint swelling since the age of 2 years. She was admitted to another hospital 2 months ago with hemoptysis, fever, and a low hemoglobin (Hb) level (4 gr/dl). On admission to our hospital, she had urticarial maculopapular rashes on the trunk and extremities, arthritis in both ankles, swelling on the dorsum of her feet, bilateral conjunctivitis, multiple micro-lymphadenopathy, and mild hepatomegaly. Laboratory test results were as follows: Hb: 8.4 g/dl, platelet count: 684,000/mm^3^, erythrocyte sedimentation rate (ESR): 78 mm/h (0–20), C-reactive protein (CRP): 6 mg/dl (0–0.5), complement 3 (C3): 0.68 g/l (0.88–2.01), and the perinuclear anti-neutrophil cytoplasmic antibody (pANCA) test was positive. The urinalysis, renal function tests, antinuclear antibody (ANA), and anti-double-stranded DNA (anti-dsDNA) tests were all normal. A skin biopsy revealed the presence of leukocytoclastic vasculitis (LCV) with fibrinoid degeneration in some vessel walls, and colchicine treatment was started; subsequently, steroid therapy was added with a probable diagnosis of microscopic polyangiitis (MPA). Her symptoms resolved within 2 weeks. Subsequently, the treatment regimen was augmented with the addition of cyclophosphamide (CYC) per oral (po) due to the development of new skin lesions. After a period of 5 months, the patient was switched to azathioprine (AZA) for the next 6 months. Subsequently, there was a 2-year interval during which she did not attend control visits, and at that time, she was taking colchicine and low-dose steroid treatment. When she was at 5.5 years of age, she was readmitted with a similar set of symptoms, mild anemia, mild acute phase reactant (APR) elevation, and hypocomplementemia. C1q (complement 1q) was undetectable in serum, and IgG (immunoglobulin) anti-C1q antibodies were 18.9 U/ml (reference range 0–10). Additionally, microscopic hematuria, mild proteinuria (9.7 mg/m^2^/h), and positive ANA results (1/300) were documented. A diagnosis of HUVS was made, etanercept (ETN) and enalapril were added to the steroid therapy, and significant improvement was observed. After 8 months, anti-dsDNA titers increased to 606 IU/ml (N: 0–40), and 24-h urine protein was detected as 33 mg/m^2^/h. Renal biopsy showed the presence of class IIA lupus nephritis with a full-house immunofluorescence staining pattern. The dosage of steroids was increased, and the first course of rituximab (RTX) was given. At age 8, the patient presented with fever, hematuria, rash, joint pain, and worsening proteinuria. A second biopsy revealed class IIIA lupus nephritis with crescents (activity index 7, chronicity index 1), and the second course of RTX was given. At age 10, proteinuria was 22 mg/m^2^/h, and a third course of RTX was given. Meanwhile, a homozygous frameshift mutation, c.289_290 delAC, in DNASE1L3 was identified through the use of a genome-wide SNP (single-nucleotide polymorphism) genotyping method, and hydroxychloroquine (HCQ) was started. At the age of 11, anterior uveitis was diagnosed, and methotrexate (MTX) was given; due to an inadequate clinical response, adalimumab was added. Treatments were discontinued after 2 years but were restarted when uveitis recurred a year later. MTX and adalimumab were continued until the age of 15. At age 17, routine follow-up revealed hematuria, proteinuria, and elevated anti-dsDNA levels. A third biopsy was performed which revealed class III lupus nephritis (activity index 4, chronicity index 2), and the fourth course of RTX with high-dose steroids was administered. At age 19, macroscopic hematuria and worsening rashes indicated active disease, and RTX treatment (fifth course) was repeated, and symptoms resolved. At the most recent follow-up appointment, she is at 22.5 years of age with no symptoms. She is currently on 2.5 mg/day of steroid and exhibits proteinuria of 3.6 mg/m^2^/h over a 24-h period. As illustrated in Fig. 1, the patient’s comprehensive treatment history is outlined in detail.

### Case 2

One year after the initial admission of the index case, her 2.5-year-old sister was admitted with rash, injection of the conjunctivae, leg pain, and swelling of the feet. A physical examination revealed macular rash, bilateral conjunctival hyperemia, multiple peripheral micro-lymphadenopathy, swelling, and erythema on both feet. Laboratory test results were as follows: Hb 11.3 g/dl, platelet count 599,000/mm^3^, ESR 40 mm/h, and CRP 1.84 mg/dl. The urinalysis, renal function tests, ANA, and anti-dsDNA and ANCA results were normal. Skin biopsy showed LCV. A course of steroid and colchicine treatment was initiated in accordance with the diagnosis of unclassified vasculitis. The patient’s symptoms resolved entirely. At the age of 4.5 years, she presented with similar symptoms, and hypocomplementemia was detected.

**Fig. 2:**
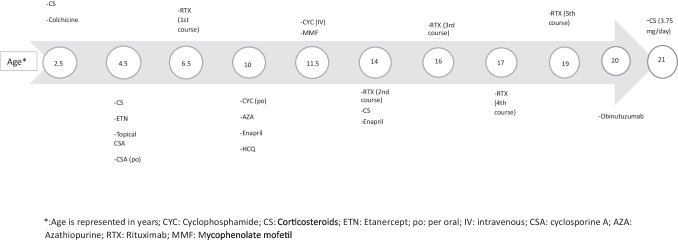
Treatment history of Case 2

Additionally, the concentration of IgG anti-C1q antibodies was 12.2 U/ml (0–10), and the C1q levels were found to be extremely low. A diagnosis of HUVS was made in conjunction with her sister. The patient was initiated on ETN, but treatment was discontinued due to lack of response. Consequently, the dosage of steroids was increased to 2 mg/kg/day. An ophthalmological examination revealed limbitis, and topical cyclosporine (CSA) drops were initiated. Additionally, oral CSA was initiated to address the systemic manifestations. At 6.5 years of age, in view of the persistence of rashes and ocular findings, taking into account that the patient’s sister benefited from RTX, the first course of RTX treatment was administered. The treatment was unsuccessful in eliciting a response. At the age of 7.5 years, a renal biopsy was conducted due to the presence of ANA positivity with proteinuria at the nephritic level, and focal segmental necrosis, focal segmental endocapillary proliferation, and GN with a vasculitic etiology were determined. The dosage of steroids was increased, and enalapril was started resulting in the attainment of clinical remission. Meanwhile, a homozygous frameshift mutation, c.289_290 delAC, in DNASE1L3 was identified in conjunction with her sister. At the age of 10, she underwent a second renal biopsy due to nephrotic proteinuria and was identified to have focal segmental necrotizing GN, so she was started on oral CYC for 3 months and then switched to AZA. HCQ was also added. The patient achieved remission. At the age of 11.5 years, a third kidney biopsy was performed owing to worsening proteinuria and showed the presence of IgG-dominant immunocomplex deposition in glomeruli, which did not reach the level of a full house. Furthermore, vascular alterations were identified, and the patient was administered a total of ten doses of intravenous CYC, and then, mycophenolate mofetil (MMF) was started. At 14 years of age, on routine follow-up, 69 mg/m^2^/h proteinuria was detected, and the fourth renal biopsy revealed GN with focal segmental fibrinoid necrosis and segmental sclerosis in glomeruli. Her steroid dose was increased, and the second course of RTX treatment was given. The patient exhibited no indications of activation (at 16 and 17 years of age); however, it was observed that CD19 and CD20 suppression had been lifted during the patient’s routine follow-up. Consequently, the patient was scheduled and administered two courses (third and fourth) of RTX treatment. During these visits, the patient’s proteinuria levels were recorded as 7 mg/m^2^/h and 9.6 mg/m^2^/h respectively. At the age of 19, the patient was referred to the otorhinolaryngology clinic due to a history of progressive hearing loss. Upon examination and audiometry, a severe sensorineural hearing loss was observed in the right ear. Due to both persistent proteinuria (11.2 mg/m^2^/h) and hearing loss, she was scheduled to receive the fifth course of RTX. She experienced anaphylaxis during the administration of the third dose of RTX, and a desensitization protocol was attempted, but discontinued due to the recurrence of anaphylaxis. At 20 years of age, obinutuzumab, another anti-CD20 monoclonal antibody, was given to the patient at 2-week intervals (750 mg/m^2^/dose, two doses). At the most recent follow-up visit, she is 21 years of age and reports no active symptoms. She is currently undergoing treatment with 3.75 mg of steroids per day and exhibits a proteinuria level of 4.3 mg/m^2^/h over a 24-h period. The patient’s comprehensive treatment history is outlined in detail in Fig. 2.

**Fig. 3:**
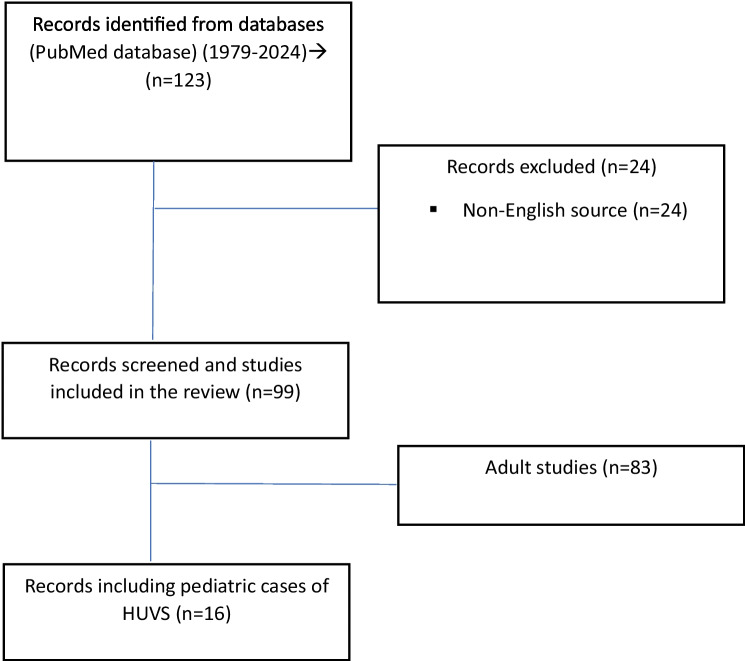
Flowchart for original English studies found in the PubMed database (https://pubmed.ncbi.nlm.nih.gov) with the query: “hypocomplementemic urticarial vasculitis syndrome”

A literature analysis was conducted by searching the PubMed database using the term “hypocomplementemic urticarial vasculitis syndrome”. Of the 123 articles identified between 1979 and 2024, only those written in English were subjected to evaluation, resulting in the exclusion of 24 articles from the assessment. After the exclusion of adult cases, encompassing a total of 16 articles that reported on cases of pediatric HUVS were included (Fig. 3) [8–23].

A total of 28 cases of pediatric HUVS were identified in the literature, including our two cases and the deceased sibling of our cases. An exhaustive evaluation of the patients’ demographic and clinical characteristics was conducted, encompassing their age at diagnosis, gender, pertinent clinical findings, and organ involvements. Renal biopsies, when available, were also analyzed. Furthermore, the laboratory anti-C1q antibody and DNASE1L3 mutation positivity were assessed, along with the treatments received by the patients and the total follow-up period. The comprehensive data set is presented in Table 1.

**Table 1:** Comprehensive data set

	Reference (year)	Age at diagnosis	Sex	Clinical presentation	Renal manifestations	Kidney biopsy	Anti-C1q antibody	DNASE1L3 Deficiency	Treatment	Clinical follow-up	Total follow-up time (years)
1	Waldo et al. [9](1985)	16	M	Recurrent urticaria, angioedema, HT	Severe hypertensive GN	MPGN	−	UA	CS(PO)	ESRD	NR
2	Martini et al. [10] (1994)	14	M	Arthritis/arthralgia, eye involvement	Proteinuria, hematuria, rapidly progressive GN	MesPGN	+	UA	CS (PO, IV), CYC (PO, IV)	ESRD	NR
3	Martini et al. [10] (1994)	15	M	Arthritis/arthralgia, eye involvement, angioedema, pulmonary hemorrhage	Hematuria	UA	+	UA	CS (PO, IV), CYC (IV), AZA	Maintenance hemodialysis	NR
4	Renard et al. [11] (1998)	13	M	Urticaria, arthritis, episcleritis, hemoptysis with intrapulmonary hemorrhage	Macroscopic hematuria, nephrotic syndrome, renal failure	MesPGN with cellular and fibrous crescents	UA	UA	CS (PO, IV), CSA, AZA	Recovery of renal function with mild proteinuria	8
5	Cadnapaphornchai et al. [12] (2000)	11	F	Urticaria, polyarthritis	Proteinuria, hematuria	MPGN	UA	UA	CS (PO)	Improvement in proteinuria with treatment	9
6	De Amicis et al. [13] (2002)	12	F	Urticaria, fever, arthritis/arthralgia	Proteinuria, hematuria	UA	UA	UA	CS	Cutaneous lesions resolved with treatment	3 months
7	Yamazaki et al. [14] (2009)	4	F	Angioedema, urticaria, episcleritis, conjunctivitis, Raynaud phenomenon, livedo reticularis, painless oral ulcers, arthritis, fever	Proteinuria	Class IV lupus nephritis	UA	UA	CS, HCQ, CYC (IV), IVIG	In remission	3
8	Özçakar et al. [15] (2013)	6	F	Recurrent abdominal pain, recurrent urticaria, fever, fatigue, lymphadenopathy	GN	Crescentic GN	UA	+	CS (IV, PO), AZA, CYC	Died 10 months later	10 months
9	Özçakar et al. [15] (2013) (Case 1)	6	F	Recurrent urticaria, arthritis/arthralgia, conjunctivitis, uveitis, lymphadenopathy	Proteinuria, hematuria	Class III lupus nephritis	+	+	CS (IV, PO), colchicine, etanercept, HCQ, AZA (PO), CYC (PO), RTX (IV), MTX, adalimumab	SLE and HUVS are in remission	22
10	Özçakar et al. [15] (2013) (Case 2)	4.5	F	Recurrent urticaria, lymphadenopathy, uveitis, conjunctivitis, arthritis/arthralgia	Nephrotic proteinuria, macroscopic hematuria	Focal segmental necrotizing glomerulonephritis	+	+	CS (IV, PO), colchicine, etanercept, HCQ, AZA (PO), CYC (IV), RTX (IV), MMF, obinutuzumab (IV)	In remission	20
11	Özçakar et al. [8] (2013)	7.5	F	Recurrent abdominal pain, recurrent urticaria, fever, fatigue, lymphadenopathy, arthritis/arthralgia	GN	Class II lupus nephritis	UA	+	NR	SLE and HUVS are in remission	13
12	Özçakar et al. [8] (2013)	3.5	F	Recurrent abdominal pain, recurrent urticaria, fever, fatigue, arthritis/arthralgia	GN	Class II lupus nephritis	UA	+	NR	Died due to active SLE crisis	6
13	Al Mosawi et al. [16] (2013)	8	M	Recurrent urticaria, fever, fatigue, arthritis/arthralgia, conjunctivitis, episcleritis, lymphadenopathy	Proteinuria	Diffuse mesangial cell hyperplasia and sclerosed glomeruli	−	UA	CS (IV), MMF	Revealed good response to treatment	3
14	Pasini et al. [17] (2014)	9	F	Abdominal symptoms (cramps, nausea, and diarrhea), conjunctivitis, recurrent urticaria, arthritis/arthralgia	Hematuria	Mesangial glomerulonephritis with membranous features	+	UA	CS (PO), MMF, Dapsone	Skin lesions resolved, hematuria persisted	18 months
15	Pasini et al. [17] (2014)	9	F	Fever, recurrent urticaria, arthritis/arthralgia, episcleritis	Hematuria, proteinuria	Mesangial glomerulonephritis associated with focal necrotizing small-vessel vasculitis	+	UA	CS (PO), CYC (IV), AZA	In remission	5
16	Pasini et al. [17] (2014)	12	F	Recurrent urticaria, arthritis/arthralgia, abdominal pain, conjunctivitis	Hematuria, proteinuria	GN with intense mesangial, endo- and extracapillary proliferation with initial tubular atrophy	+	UA	CS (PO, IV), CSA, colchicine, anakinra, HCQ, dapsone, AZA, CYC (IV)	In remission	5
17	Jung et al. [18] (2017)	15	M	Recurrent urticaria, arthritis/arthralgia	Proteinuria	Membranous nephropathy	+	UA	CS (PO, IV), AZA, HCQ, telmisartan, dapsone, tacrolimus	Ongoing experience with flares	16 months
18	Al Hermi et al. [19] (2017)	6	M	Recurrent urticaria, arthritis/arthralgia, conjunctivitis, abdominal pain	Proteinuria, hematuria	MesPGN	+	UA	CS (PO, IV), AZA	In remission	NR
19	Ranalli et al. [20] (2019)	9	NR	Arthritis/arthralgia, cholecystitis	NR	Lupus nephritis	+	+	NR	NR	NR
20	Ranalli et al. [20] (2019)	3	NR	Arthritis/arthralgia, cholecystitis, pulmonary vasculitis, ocular involvement	NR	Lupus nephritis	+	+	NR	NR	NR
21	Ranalli et al. [20] (2019)	14	NR	NR	NR	Lupus nephritis	+	+	NR	NR	NR
22	Corthier et al. [21] (2022)	12	F	Recurrent urticaria, arthritis/arthralgia, fever, conjunctivitis, respiratory disease	Proteinuria	MPGN	UA	UA	CS (PO, IV), CYC (IV), RTX (IV), MMF, plasma exchange	ESRD, kidney transplantation without HUVS relapse	NR
23	Corthier et al. [21] (2022)	5	M	Recurrent urticaria, fever	Proteinuria, hematuria	MPGN	UA	UA	CS (PO, IV)	NR	NR
24	Tusseau et al. [22] (2022)	11	F	Recurrent urticaria, arthritis/arthralgia	GN	Lupus nephritis	UA	+	CS, MMF, HCQ, tacrolimus	In remission	6 months
25	Tusseau et al. [22] (2022)	15	F	Recurrent urticaria, arthritis/arthralgia, myalgia	NR	UA	UA	+	CS, HCQ, AZA	In remission	2 months
26	Tusseau et al. [22] (2022)	1.5	M	Recurrent urticaria, arthritis/arthralgia, abdominal pain, fever, anemia, poor weight gain, vomiting	NR	UA	+	+	CS, AZA, CYC, MMF	Has active IBD	9
27	Tusseau et al. [22] (2022)	4	F	Recurrent urticaria, arthritis/arthralgia, abdominal pain, fever	Hematuria, proteinuria, HT	Immune complex–mediated glomerulonephritis,	+	+	NR	Kidney transplantation	NR
28	Lin et al. [23] (2024)	14	M	Urticaria, arthritis/arthralgia, respiratory distress, abdominal pain, fever	NR	UA	+	UA	NR	NR	NR

*F* female, *M* male, *HT* hypertension, *UA* unattended, *NR* not reported, *GN* glomerulonephritis, *MPGN* membranoproliferative glomerulonephritis, *CS* corticosteroids, *PO* per oral, *ESRD* end-stage renal disease, *MesPGN* mesangial proliferative glomerulonephritis, *IV* intravenous, *IVIG* intravenous immunoglobulin, *CYC* cyclophosphamide, *AZA* azathioprine, *RTX* rituximab, *MTX* methotrexate, *HCQ* hydroxychloroquine, *MMF* mycophenolate mofetil, *SLE* systemic lupus erythematosus, *HUVS* hypocomplementemic urticarial vasculitis syndrome, *IBD* inflammatory bowel disease

## Discussion

In this report, we presented long-term follow-up (20 years) and treatment of two siblings with HUVS. Moreover, a literature review regarding clinical findings and treatment of all pediatric patients (*n* = 28) was summarized. The follow-up period was not documented in 11 patients (39%), and the mean follow-up period was 6.23 ± 6.64 years in the remaining ones. Except for our patients, one patient was followed up for approximately 13 years, and six patients had a follow-up period of 5–9 years. Two patients died, and five developed end-stage renal disease (ESRD). The prognosis appears to be poor over the long term.

The clinic of our patients exhibited both similarities and differences. Our two cases both presented with constitutional, skin, joint, eye, and renal findings. One of our patients developed anterior uveitis (treated with MTX and adalimumab), while the other developed limbitis (treated with topical/oral CSA). Both of our patients had renal involvement, one with lupus nephritis and the other with vasculitic renal involvement. The patient with lupus nephritis was treated with RTX. The patient with vasculitic renal involvement was treated with CYC, MMF, RTX, and obinutuzumab. Although the disease could be controlled for a short time with CYC and MMF treatments, it exacerbated, and the patient also benefited from RTX treatment. These two patients had the longest follow-up period in the literature. The third sick sibling also had renal involvement, but exhibited severe crescentic and necrotizing GN. She was treated with CS, CYC, and AZA in another hospital, followed up for approximately 10 months and died due to sepsis [15]. It is evident that CS and RTX emerge as the most efficacious treatment modalities in our patients.

As illustrated in Table 1, 54% of the pediatric HUVS patients were female, and the age at diagnosis ranged from 1.5 to 16 years. The most common clinical symptoms were recurrent arthritis/arthralgia and urticaria. Eye involvement was noted in 46%, pulmonary involvement in 18%, and renal involvement in 79% of the patients. Renal biopsies showed lupus nephritis in 8 (36%), mesangioproliferative GN (MesPGN) in 7 (32%), and membranoproliferative glomerulonephritis (MPGN) in 4 patients (18%). Anti-C1q antibody positivity was reported in 15 patients (54%), while DNASE1L3 deficiency was reported in 12 patients (43%). Both of the two patients who died had DNASE1L3 deficiency. Treatment modalities were given in 21 patients. All were treated with CS; AZA was administered to 11 patients, CYC to 10 patients, MMF to 6 patients, and colchicine to 6 patients, and RTX was administered to 3 patients. A wide range of treatments were explored, including dapsone, intravenous immunoglobulin (IVIG), HCQ, ETN, adalimumab, MTX, obinutuzumab, anakinra, telmisartan, tacrolimus, and plasma exchange therapies. End-stage renal failure developed in five patients (18%), and two patients (7%) underwent renal transplantation. Meanwhile, two patients (7%), both with renal involvement (one with crescentic GN and the other with lupus nephritis), died. In a total of nine patients (32%), all observed findings were found to be in remission. However, 11 of 28 patients had no follow-up, and the majority of the remaining cases had short follow-up periods. Therefore, we could not exactly know what happened to these patients in the long term.

The prevalence of renal involvement in patients with HUVS varies considerably between different cohorts, with estimates ranging from 14 to 50%. However, there is currently no consensus on the precise definition of this condition [4, 21, 24]. Ion et al. provided an exhaustive review of the literature on HUVS cases reported from 1976 until 2020. A total of 60 patients (11 of them pediatric) were identified, out of which 52 patients underwent a percutaneous kidney biopsy. The most common renal manifestation was hematuria accompanied by proteinuria, while one-third exhibited abnormal kidney function at the time of presentation [25]. The most frequent glomerular pattern of injury was MPGN (35%), followed by MesPGN (21%) and membranous glomerulonephritis (MGN) (19%). As is the case with other forms of systemic vasculitis, renal involvement is associated with a poorer prognosis [26]. In a separate study conducted by Corthier in 2022, 12 cases were presented for comparison with 36 HUVS patients with renal involvement as documented in the literature. Majority of them were adult patients [21]. Of the 48 patients, 71% presented with proteinuria, 27% with nephrotic syndrome, 54% with microscopic/macroscopic hematuria, and 38% with acute kidney injury. Renal biopsies revealed the following: 23 cases of MPGN, 3 cases of MGN, 5 cases of lupus-like GN, 5 cases of crescentic GN, and 2 cases of interstitial nephritis. Interestingly, in our literature review, approximately 80% of the pediatric patients had renal involvement, and lupus nephritis was the most frequent biopsy finding.

Systemic glucocorticoids represent the primary treatment option for HUVS. However, in the majority of the patients, corticosteroid-sparing immunosuppressive agents were required, especially in patients with systemic involvement. A variety of therapeutic agents have been employed, including AZA, CYC, MMF, CSA, and MTX [27]. In cases of pulmonary and/or renal involvement, the use of immunosuppressive drugs is strongly recommended [28]. Corthier et al. reported 12 adult patients with HUVS and renal involvement; biopsies showed MPGN in eight patients, pauci-immune crescentic GN in two patients, necrotizing vasculitis in one patient, and interstitial nephritis in one patient [21]. Corticosteroids were prescribed for all patients experiencing renal flares associated with HUVS. In some cases, corticosteroids were continued for several years. An additional immunosuppressive therapy was prescribed for nine patients, and RTX was used in five of them (one as a first-line therapy, three after corticosteroids, and one after CYC) [21]. As evidenced by the findings of this study, RTX was used mostly as a second- or third-line therapy, but could be proposed as a corticoid-sparing agent in relapsing forms of HUVS. A patient with HUVS and SLE who had previously demonstrated resistance to MMF, high-dose methylprednisolone, and IVIG was successfully treated with RTX [29]. In an extensive literature review of 60 patients with HUVS and renal involvement, mentioned above, the most commonly employed immunosuppressive regimens (86% of cases) included corticosteroids in high doses. The second most commonly used drug was CYC (34%), followed by AZA (22%), MMF (15%), and CSA (8%). On occasion, plasma exchange (8%) and IVIG (7%) were also prescribed [25]. As evidenced by the extant literature, the optimal treatment for pediatric and adult HUVS patients remains unclear.

The two sisters we present here have received many treatments since they were very young, including steroids, colchicine, HCQ, MTX, CSA, CYC, MMF, ETN, and adalimumab for organ involvement such as the skin, eye, and kidney. However, both benefited significantly from RTX treatment, which was given after the development of kidney involvement. The patient who developed lupus nephritis achieved long-term remission. The other patient’s clinical findings partially resolved, and intermittent RTX treatment was planned but could not be administered due to the development of anaphylaxis. At that time, she was given obinutuzumab as an alternative treatment and has been followed in remission for approximately 1 year.

The significant impact of the DNASE1L3 mutation on the chronic and severe course of the two sisters’ disorder, which has persisted for around 20 years, is undoubted. In 28 pediatrics HUVS cases till now, DNASE1L3 deficiency was reported in 12 patients (43%). DNASE1L3 encodes a unique extracellular enzyme with the ability to digest chromatin and microparticle DNA released from apoptotic cells. The accumulation of microparticles containing extracellular DNA can induce B cells to produce autoantibodies against C1q or DNASE1L3, which also contribute to the development of SLE nephritis [30]. The good response of these patients, especially those with renal involvement, to CD-20 monoclonal antibody therapy targeting B cells can be explained by the role of B cells in pathogenesis. DNASE1L3 deficiency has been shown to be clearly associated with a lupus phenotype [31].

HUVS is a rare multisystem immune complex–mediated disease for which there is currently a lack of understanding, and most studies are case studies of a small number of patients. A review of the literature shows 28 pediatric patients diagnosed with HUVS since its definition. The small number of patients has posed a significant challenge in conducting studies and in the present literature review.

## Conclusion

The treatment of HUVS presents a significant challenge due to the variable clinical spectrum, the lack of approved medications, and the absence of evidence-based guidelines. Treatment is also determined more by the clinical findings. We have presented a long-term follow-up of patients (over 20 years) with familial HUVS. Our long-term observation indicates that B-cell depletion with CD20 monoclonal antibody treatment seems to be more efficacious than other forms of therapy.

The present study has contributed to the emphasis on this issue by revealing the necessity for further studies in the future on the etiology, clinical course, and treatment approaches of pediatric HUVS. Hence, larger cohort studies are required to investigate genetic and environmental factors contributing to the etiology, and large-scale clinical trials are needed to evaluate the efficacy of different treatment strategies.

## Data Availability

Data are available to the public when a reasonable request is made.
